# Preparation and Identification of Per a 5 as a Novel American Cockroach Allergen

**DOI:** 10.1155/2014/591468

**Published:** 2014-02-23

**Authors:** Ji-Fu Wei, Haiwei Yang, Dongning Li, Peisong Gao, Shaoheng He

**Affiliations:** ^1^Allergy and Clinical Immunology Research Centre, The First Affiliated Hospital of Liaoning Medical University, Jinzhou, Liaoning 121001, China; ^2^Research Division of Clinical Pharmacology, The First Affiliated Hospital of Nanjing Medical University, Nanjing, Jiangsu 210029, China; ^3^Department of Urology, The First Affiliated Hospital of Nanjing Medical University, Nanjing, Jiangsu 210029, China; ^4^Johns Hopkins Asthma & Allergy Center, Baltimore, MD 21224, USA

## Abstract

Glutathione S-transferase (GST) from various arthropods can elicit allergic reactions. In the present study, Per a 5, a GST, was cloned from American cockroach (CR) and expressed in both baculovirus-infected insect cell (iPer a 5) and *E. coli* expression (bPer a 5) systems. The secondary structures were predicted to be 45.93 and 8.69% of **α**-helix **β**-sheets in iPer a 5 and 42.54 and 8.49% of **α**-helix and **β**-sheets in bPer a 5, respectively. It is found that 4 out of 16 (25%) sera from American CR allergy patients reacted to both bPer a 9 and iPer a 9 as assessed by ELISA and Western blotting analysis, confirming that Per a 5 is not a major allergen of American CR. Induction of upregulated expression of CD63 and CCR3 on passively sensitized human basophils (sera from American CR allergy patients) by approximately up to 4.5- and 3.2-fold indicates that iPer a 5 and bPer a 5 are functionally active. Recombinant Per a 5 (rPer a 5) should be a useful tool for studying and understanding the role of Per a 5 in CR allergy.

## 1. Introduction

CR allergy has been recognized as important IgE-mediated type I hypersensitivity since 1964 [1]. It is associated with the development of asthma and recognized as a risk factor for emergency room admission of asthmatic patients, especially among inner city children living in low-income houses infested with cockroaches [2]. In China, totally 25.7% of allergy patients are skin prick test (SPT) positive to the American CR (*Periplaneta americana*, Per a) and 18.7% SPT positive to the German CR (*Blattella germanica*, Bla g) [3].

American CR, German CR, and smoky brown CR (*Periplaneta fuliginosa*) are the dominant indoor CR species which cause allergy among human population worldwide [2]. Twenty-two immunoglobulin E (IgE) binding components including the proteins of 23, 28, 35, 38, 40, 49, 72, 78, and 97 kDa were identified as major allergens in American CR [4], but only Per a 1 [5], Per a 2 [6], Per a 3 [7], Per a 4 [8], Per a 7 [9], Per a 9 [10], and Per a 10 [11] are characterized. For example, Per a 1 is an isoallergen with 5 isoforms reported so far, that is, Per a 1.0101–Per a 1.0105. Per a 1 is a major American CR allergen as it binds to IgE in the sera of 90–100% of CR allergic subjects [4]. Per a 2 is an inactive aspartic protease found in the American CR digestive tract and feces [5]. Per a 5 belongs to GSTs, but the allergenicity of the GST homologues in American CR has not been reported. Since the GST of German CR (Bla g 5) is a major cockroach allergen, which induces specific IgE expression in 30 to 71% of CR allergy [12–14], we anticipate that Per a 5 is likely a major allergen of American CR and investigate the potential allergenicity of it in the present study.

Two GST homologue (Per a 5) genes are available in the Genbank (Accession: AY792949 and AEV23867), which provides the possibility of producing recombinant Per a 5 (rPer a 5) in large amounts to study its role in allergic reactions. Many, but not all allergens expressed from cDNA have shown a considerable IgE binding reactivity that seems to be comparable to their natural counterparts. The majority of these recombinant allergens are produced in* E. coli*, but, unfortunately, the amount and/or reactivity is sometimes reduced when the allergen is purified and subjected to immunological and biochemical assays [15]. To overcome some of these problems, eukaryotic expression systems such as yeast and baculovirus in insect cells have been used [16]. The aim of the present study is to generate rPer a 5 by using eukaryotic (baculovirus-infected insect cells) and prokaryotic (*E. coli*) expression systems and characterize its biochemical and immunologic properties.

## 2. Materials and Methods

### 2.1. Ethics Statement

The study protocol was approved by the Ethical Committee of the First Affiliated Hospital of Nanjing Medical University. Written informed consent for the use of blood samples was obtained from all participants before study entry according to the declaration of Helsinki.

### 2.2. Patients and Samples

A total of 16 allergic rhinitis patients with positive SPT (allergens were supplied by ALK-Abelló, Inc., Denmark) and positive serum IgE test to American CR extract (by using Immuno-CAP assay (Pharmacia Diagnostics AB, Uppsala, Sweden)) and 6 healthy controls (HC) were recruited in the study. Serum (4 mL) from peripheral venous blood was collected from each patient and HC for Western blot analysis.

### 2.3. Cloning of cDNAs Encoding Full Length of Per a 5 Gene

Total RNA was isolated from adult female CR reared at our institute by using TRIzol reagent (Invitrogen, Carlsbad, CA, USA). Total RNA was quantified by measuring absorbance ratios at 260/280 nm. The cDNA was prepared by reverse transcription using a commercial RNA-PCR kit according to the manufacturer's instruction (TaKaRa Biotech Co. Ltd., Dalian, China). For each reaction, 1 *μ*g of total RNA was reversely transcribed using oligo-d (T). The cDNAs encoding Per a 5 were amplified by PCR using primers based nucleotide sequence of Per a 5 gene (AY792949 and AEV23867; forward: 5′-ATGACCATCGACTTCTACTA-3′; reverse: 5′-TCACTTCTTGGCGAGGTTAT-3′). PCR condition was 95°C/5 min (one cycle), 95°C/1 min, 52°C/1 min and 72°C/1 min (30 cycles), and 72°C/5 min (one cycle). The purified PCR product was cloned into apMD18-T vector (TaKaRa Biotech Co. Ltd., Dalian, China) before being transformed into *Escherichia coli* strain DH5*α*. The inserts were sequenced on an ABIprism 377 DNA sequencer (Applied Biosystems, Foster, CA, USA). DNA sequence data were translated to amino acid sequence by Show Translation tool in SMS software package (http://www.bioinformatics.org/SMS/). The glycosylation motifs of Per a 5 were predicted by using NetNGlyc 1.0 Server (http://www.cbs.dtu.dk/services/NetNGlyc).

### 2.4. Expression and Purification of Per a 5 in Baculovirus-Infected Insect Cells

The Per a 5 gene was subcloned into pFastBac1 vector (Novagen, Madison, WI, USA) using *EcoR* I and* Sal I* sites and the resulted construct was transformed into *E. coli *strain DH10Bac to generate recombinant bacmid. The positive colonies were selected and followed by PCR identification. The recombinant bacmid was transfected into Sf-9 cells by using Cellfectin (Invitrogen Corporation, Carlsbad, USA) and incubated in SF-900II liquid medium (Invitrogen Corporation, Carlsbad, USA) for 5 days at 27°C until the cells got swollen. The supernatant was collected as P1 viral stock. P2 viruses were amplified for later infection. A total of 500 mL of Sf-9 cells were infected by P2 viruses and harvested at 72 h. The cells were lysed against 50 mM Tris-HCl with 300 mM NaCl and 5% glycerol. The supernatant was loaded on Ni-NTA column (Genscript, Nanjing, China), washed with running buffer containing 50 mM Tris-HCl, 300 mM NaCl, and 5% glycerol (pH 8.0) and eluted with elution buffer containing 50 mM Tris-HCl, 300 mM NaCl, 250 mM imidazole, and 5% glycerol (pH 8.0). The eluted fractions were obtained and identified as Per a 5 (iPer a 5). The purified iPer a 5 was dialyzed in carbonate-bicarbonate buffer (0.05 M, pH 9.6) for further investigation. The concentration of iPer a 5 was determined by using a Coomassie Plus assay kit with BSA as standard (Thermo Scientific Pierce, Rockford, IL, USA).

### 2.5. Expression and Purification of Per a 5 in *E. Coli*

The Per a 5 gene was subcloned into pCold II vector (TaKaRa Biotech Co. Ltd., Dalian, China) using *Nde I* and *Xba I* sites and verified by DNA sequencing. The recombinant pCold II-Per a 5 plasmid was transformed into *E. coli *Origami host strain. A colony of the selected transformed *E. coli *Origami on an overnight LB-ampicillin agar plate was inoculated into 5 mL of LB-ampicillin broth and incubated at 37°C overnight. For IPTG optimization, the overnight culture was added into fresh LB media in a ratio of 1 : 100. Once cell density reached the optical density at *A*_600 nm_ (OD_600_), the cells were incubated with 0.1, 0.5, and 1 mM IPTG, respectively, at 15°C overnight. Expression of the target protein was analyzed by SDS-PAGE. For scale-up expression, 40 mL of the culture was inoculated into 2 L of fresh LB-ampicillin broth and incubated at 37°C until OD_600_ reached 0.6. IPTG was added to the final concentration of 0.5 mM and the culture was incubated overnight. The bacterial cells were harvested by centrifugation at 4,000 ×g at 4°C for 20 min and were lysed in a lysis buffer by sonication at 20 kHz, 2 min pulse-on, 3 min pulse-off. Cell debris was removed by centrifugation at 12,000 ×g at 4°C for 20 min. The supernatant was loaded on the Nickel column (Genscript, Nanjing, China) as described above, and the eluted fractions were obtained and identified as Per a 5 (bPer a 5). The purified bPer a 5 was dialyzed in carbonate-bicarbonate buffer (0.05 M, pH 9.6) for further investigation. The concentration of bPer a 5 was determined by using a Coomassie Plus assay kit with BSA as standard.

### 2.6. CD Analysis of rPer a 5 Expressed in *E. Coli* and Insect Cells

Far UV CD spectra of bPer a 5 and iPer a 5 were collected on a Jasco J-810 spectropolarimeter (Japan Spectroscopic Co., Tokyo, Japan) using a 1 mm path length quartz cuvette at protein concentrations of 0.1 mg/mL. Spectra were measured from 240 to 190 nm, with a 0.5 nm resolution at a scanning speed of 50 nm/min, and resulted from averaging of three scans. All measurements were performed in 10 mM Na_2_HPO_4_, pH 7.0. The final spectra were baseline corrected by subtracting the corresponding buffer spectrum. Results were expressed as the mean residue ellipticity (*y*) at a given wavelength. The secondary structure content of bPer a 5 and iPer a 5 was calculated by using the secondary structure estimation program K2D2 [17].

### 2.7. Immunoreactivity of Human Sera with rPer a 5

A 96-well plate was coated with purified bPer a 5 and iPer a 5 at 10 *μ*g/mL in carbonate-bicarbonate buffer (0.05 M, pH 9.6) overnight at 4°C, 100 *μ*L per well. Human serum samples (1 : 20 dilution in PBS-Tween with 2% BSA) were then added to the plates for 2 h at room temperature. After IgE binding, plates were incubated with horseradish peroxidase-labeled goat anti-human IgE (1 : 2500 dilution) (KPL, Inc., MD, USA), and the color was developed with tetramethylbenzidine peroxidase substrate (Thermo Scientific Pierce, Rockford, IL, USA). The plates were read on a microplate reader at absorbance of 405 nm. The cutoff of the ELISA was calculated as the mean of the negative controls plus 2 SDs.

For competitive ELISA test, a 96-well plate was coated with American cockroach extract (10 *μ*g/mL) in carbonate-bicarbonate buffer (0.05 M, pH 9.6) overnight at 4°C. The 1 : 20-diluted pooled serum alone or preincubated with various quantities of the crude extract, iPer a 5 and bPer a 5, for 2 h was added to the plates. The color development and plate reading procedure was the same as the previous section. Crude extract of cockroaches were prepared according to the methods described previously [18] with few modifications. Briefly, 30 g of cockroaches was pulverized in liquid nitrogen. The sample was defatted in 200 mL of ethyl ether and ethyl acetate (1 : 1, by volume) and extracted with slow overhead stirring in carbonate-bicarbonate buffer (0.05 M, pH 9.6), containing 6 mM 2-mercaptoethanol and 1/1,000 volume of protease inhibitor cocktail (Shenggong, Shanghai, China) at 4°C overnight. The extract was then centrifuged at 10,000 ×g for 30 min at 4°C, and the supernatant was filtered through a 0.22 *μ*m-pore-size filter (Millipore, Bedford, USA) before use.

### 2.8. Immunoblot Analysis of IgE Reactivity

Immunoblots for detection of serum specific IgE were performed with bPer a 5 and iPer a 5 as described previously [19, 20]. bPer a 5 and iPer a 5 (5 *μ*g) were added to a SDS-PAGE (gel concentration of 15%) under reducing conditions and then transferred to nitrocellulose membranes. The nitrocellulose membranes were incubated with the sera from the patients with American CR allergy (1 : 5 in PBS-Tween with 1% BSA, 10% normal goat serum) for 90 min. Following rinsing with PBS, the membranes were incubated with peroxidase-labeled anti-human IgE monoclonal antibody. The positive protein bands were visualized by incubating the membranes with tetramethylbenzidine peroxidase substrate. Sera from 2 nonatopic subjects were used as negative controls.

### 2.9. Basophil Activation Test

Expression of CD63 and CCR3 on basophil surface has been considered as the indicator of basophil activation [21, 22]. Briefly, peripheral blood mononucleated cells (PBMC) from 20 mL blood donated by 4 healthy volunteers were separated by Ficoll-Paque density gradient and treated with 10 mL LS (a solution containing 1.3 M NaCl, 0.005 M KCl, and 0.01 lactic acid, at pH 3.9) for 2 min at 8°C. After neutralization with 12% Tris (pH 10.9), nonspecific IgE on basophils was stripped off and cells were passively sensitized with sera of the patients with American CR allergy or HC (*n* = 4, 1 in 10 dilution, 2 h at 37°C) as described previously [23]. The cells were then challenged with various concentrations of bPer a 5 and iPer a 5 for 15 min at 37°C. A goat anti-human IgE antibody (Serotec, Kidlington, UK) was used as a positive control. Anti-human CCR3-PE antibody (eBioscience Inc. San Diego, CA, USA) and anti-human CD63-FITC antibody (Invitrogen Corporation, Camarillo, CA, USA) were added to cells for 15 min at 37°C in the dark. Flow cytometry analysis of surface markers was performed at 488 nm on a FACSAria flow cytometer (Becton Dickinson, Franklin Lakes, NJ, USA) and analyzed by FACSDiva software. Basophils were gated in the lymphocyte region of the SSC or FSC pattern, and identified as a single population of cells that stained positively for CCR3-PE antibody. Upregulation of CD63 expression was determined by an increase in fluorescence in the FL-1 channel. Acquisition was terminated after 300 basophil target events. Responses were quantified as percentages of CD63 expressing basophils in a higher FL-1 region, which had been adjusted to contain 4% of basophils.

### 2.10. Statistics

Data are expressed as mean ± SEM for the indicated number of independently performed duplicated experiments. Statistical significance between means was analyzed by one-way ANOVA or the Student's *t*-test utilizing the SPSS 13.0 version. *P* < 0.05 was taken as statistically significant.

## 3. Results 

### 3.1. Cloning of cDNAs Encoding Full Length of Per a 5 Sequence

**Figure 1:**
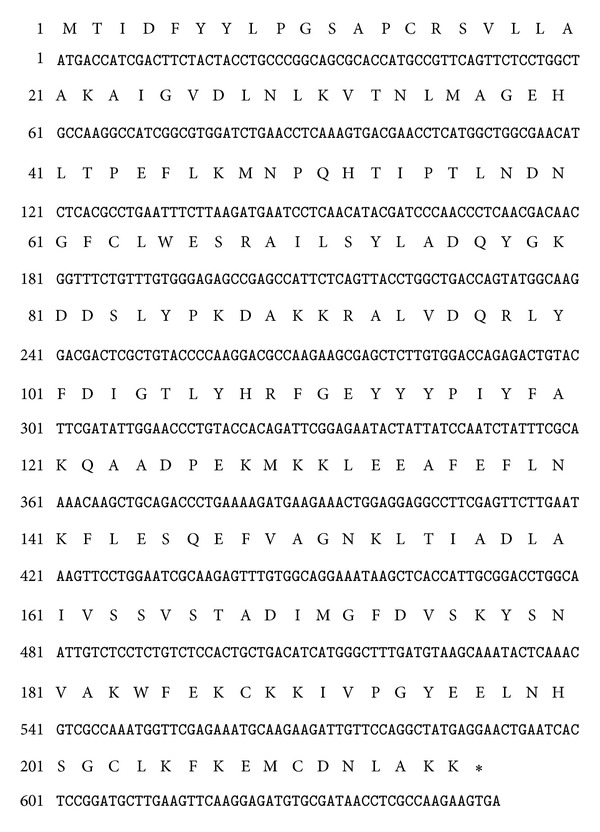
cDNA sequence encoding Per a 5 and deduced amino acid sequence. The first three bases ATG represent start codon. The last three bases indicate the stop codon.

**Figure 2:**
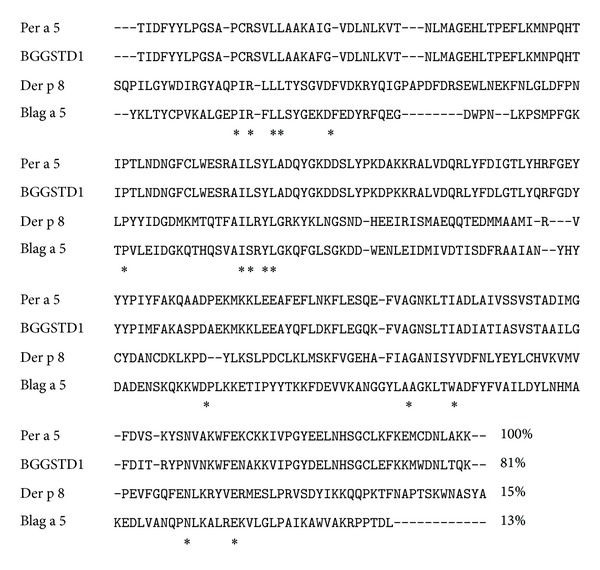
Alignment of Per a 5 with other GST allergen. Deduced amino acid sequence of Per a 5 is aligned with GST allergens. Per a 5 was cloned and sequenced in the present study. BGGSTD1 [29] and Bla g 5 [13] are two GST allergens identified in German CR, and Der p 8 is a GST allergen identified from *Dermatophagoides pteronyssinus* [30]. The identical residues in all four sequences are marked with asterisk (∗).

The cDNAs encoding Per a 5 were amplified by PCR using primers based on the nucleotide sequence of Per a 5 gene. It is a 645 bp gene and encoded 215 amino acids protein (Figure 1). The sequence identity of Per a 5 to cDNAs deposited in Genbank (Accession no. AY563004) was 100% (215/215) at protein level. Per a 5 shows 81, 15, and 13% sequence similarity to German CR allergen BGGSTD1, Der p 8, and Bla g 5 (Figure 2). One motif (198NHSG201) was predicted to be the glycosylation motif of Per a 5.

### 3.2. Expression and Purification of Per a 5 in *E. Coli*

**Figure 3:**
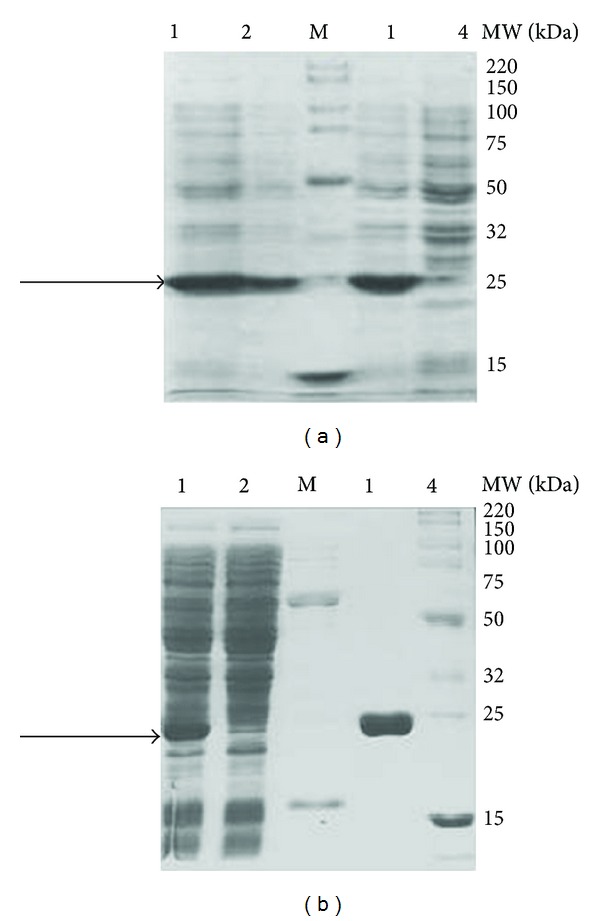
Expression and purification of Per a 5 in *E. coli*. (a) Per a 5 was induced by 0.1, 0.5, and 1 mM IPTG, respectively, and was analyzed by SDS-PAGE. Lane M: Smart Broad-Range Protein Standard (Genscript, Nanjing, China); lanes 1, 2, 3, and 4 represent lysates of cells induced with 0.5, 0.1, 1.0, or 0 mM IPTG, respectively. The arrow indicates Per a 5 protein. (b) SDS-PAGE analysis of purified Per a 5 expressed in *E. coli*. Lane M: protein standard; lane 1: the supernatant of cells lysate after centrifugation; lane 2: run through; lane 3: wash with 50 mM imidazole; lane 4: wash with 250 mM imidazole. The arrow indicates recombinant Per a 5 protein.

The Per a 5 gene was subcloned into pCold II vector and transformed into *E. coli* Origami host strain. The optimal induction condition for Per a 5 was 0.5 mM IPTG (Figure 3(a)), the concentration chosen as the final condition throughout the study. The Per a 5 protein was purified by Ni column. More than 6 mg bPer a 5 was obtained from 2 L cell culture medium. The purity of the purified Per a 5 was identified by SDS-PAGE. It showed single band with an apparent molecular weight of 25 kDa (Figure 3(b)).

### 3.3. Expression and Purification of Per a 5 in Baculovirus-Infected Insect Cells

**Figure 4:**
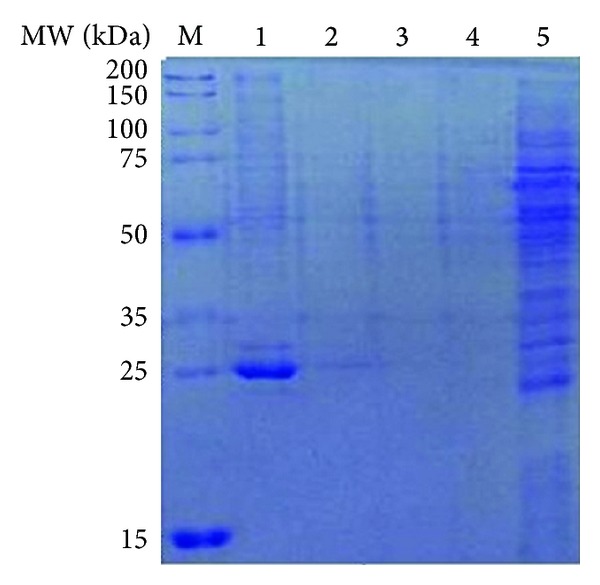
SDS-PAGE analysis of purification of Per a 5 in baculovirus-infected insect cells through Ni column. Lane M: protein standard; lane 1: elute with the elution buffer containing 250 mM imidazole; lane 2: elute with the elution buffer containing 20 mM imidazole; lane 3: elute with the elution buffer containing 50 mM imidazole; lane 4: run through; lane 5: the cytosol.

The Per a 5 encoding gene was subcloned into pFastBac1 vector and transformed into *E. coli *strain DH10Bac to generate recombinant bacmid. The recombinant bacmid was then transfected into Sf-9 cells to generate the baculovirus. The Per a 5 protein was expressed in Sf-9 cells and purified by Ni column. About 16 mg of iPer a 5 was obtained from 2 L cell culture medium. The purity of the purified Per a 5 was identified by SDS-PAGE, which showed major band with an apparent molecular weight of 25 kDa (Figure 4).

### 3.4. CD Analysis of rPer a 5 Expressed in Insect Cells and *E. Coli*

**Figure 5:**
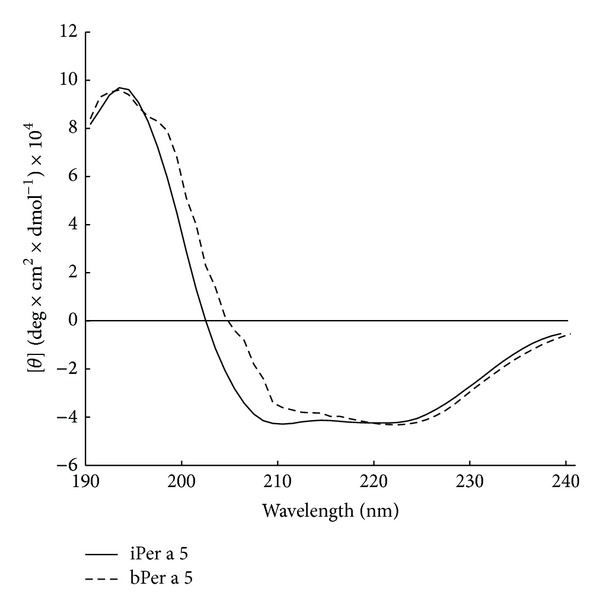
Circular dichroism analysis of recombinant Per a 5 generated from baculovirus-infected insect cells (iPer a 5) and *E. coli* (bPer a 5). The molecular ellipticities (*y*) (*y*-axes) at different wavelengths (190–240 nm, *x*-axis) are displayed for iPer a 5 and bPer a 5.

The far UV CD spectra of iPer a 5 and bPer a 5 showed similar curves with two minima at 220 and 209.5 nm and a large maximum at 192 nm, which represent characteristics of proteins with predominantly *α*-helical structure (Figure 5). Calculation of the secondary structure using the program K2D2 resulted in predicted 45.93 and 8.69% of *α*-helix and *β*-sheets in iPer a 5 and 42.54 and 8.49% of *α*-helix and *β*-sheets in bPer a 5, respectively.

### 3.5. Immunoreactivity to IgE

**Figure 6:**
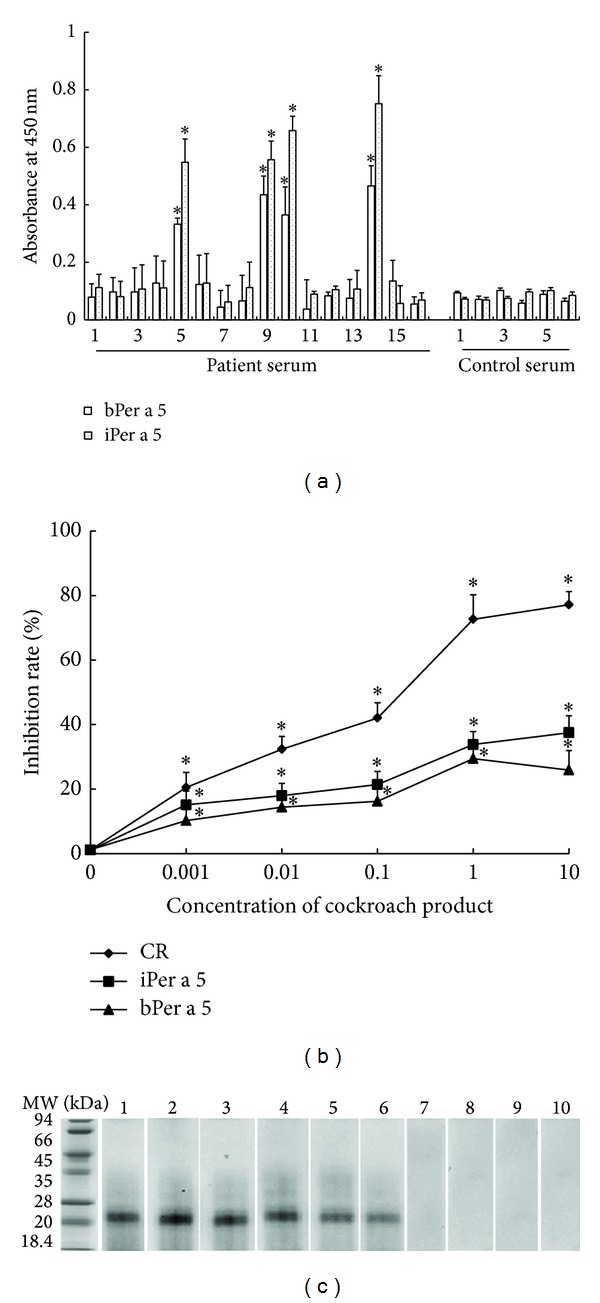
(a) Analysis of specific IgE reactivity of recombinant bPer a 5 and iPer a 5 by direct ELISA. The sera were collected from the patients with American CR allergy and healthy control subjects. The values shown are mean ± SEM for the triplicate experiments. *P < 0.05 in comparison with those of healthy control subjects. (b) Inhibition of the IgE reactivity to the cockroach extract by bPer a 5 and iPer a 5. CR = American cockroach extract. The values shown are mean ± SEM for the triplicate experiments. *P < 0.05 in comparison with the uninhibited control. (c) Western blot analysis of IgE reactivity to bPer a 5 and iPer a 5 in the sera from the patients with American CR allergy. Lanes 1–3: iPer a 5 reacted with the serum from patients 5, 9, and 10. Lanes 4–6: bPer a 5 reacted with the serum from patients 5, 9, and 10. Lanes 7-8: iPer a 5 reacted with the serum from controls 1 and 2. Lanes 9-10: bPer a 5 reacted with the serum from controls 1 and 2.

In order to determine the allergenicity of Per a 5, we examined the ability of Per a 5 to bind IgE in the sera of American CR allergy by a direct ELISA technique. Patients serum including patients 5, 9, 10, and 14 showed positive IgE reactivity to both bPer a 5 and iPer a 5. The results showed that 4 out of 16 (25%) sera from these patients reacted to both bPer a 5 and iPer a 5 (Figure 6(a)). The IgE reactivity of bPer a 5 and iPer a 5 in the sera from the Per a 5 positive patients was increased by 5.0- and 7.9-fold, respectively in comparison with the sera from HC. Moreover, competitive ELISA showed that bPer a 5 and iPer a 5 inhibited the IgE reactivity to the cockroach extract by approximately 25.4 and 35.5%, respectively (Figure 6(b)). IgE binding activity of Per a 5 in a representative group of 3 patients and two HC was assessed by Western blot and was illustrated in Figure 6(c). IgE binding bands appeared clearer with iPer a 5 than with bPer a 5. Both iPer a 5 and bPer a 5 did not react to the sera from the HC.

### 3.6. Induction of Basophil Activation by rPer a 5

**Figure 7:**
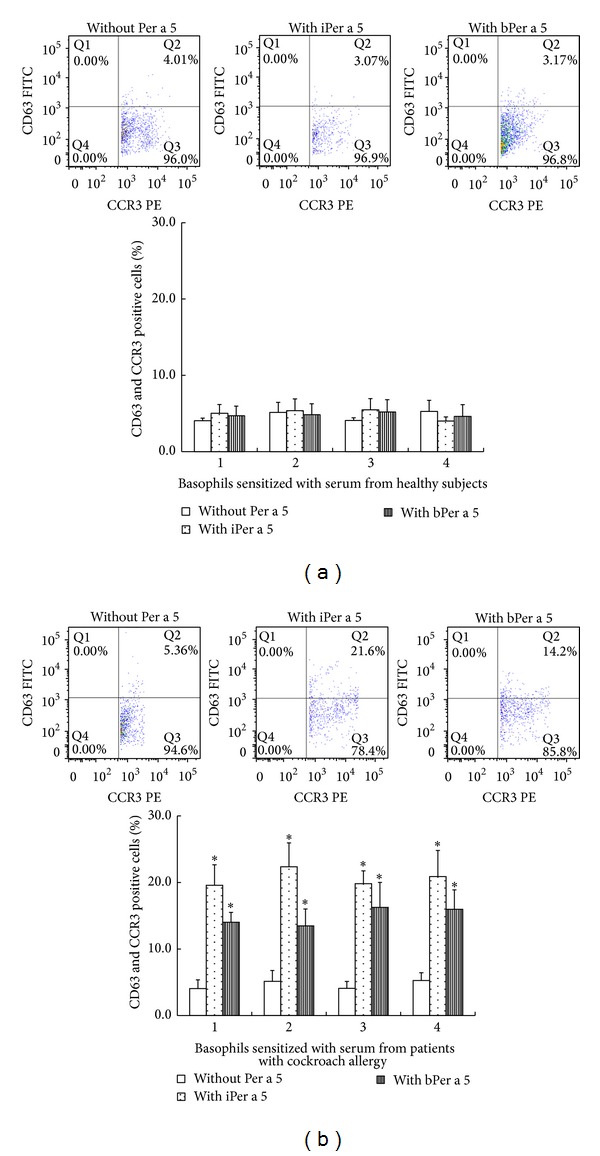
Induction of basophil activation by iPer a 5 and bPer a 5. After nonspecific IgE on basophils being stripped off, cells from each donor were passively sensitized with sera from 4 different healthy subjects (a) or from 4 different patients with American CR allergy (b) and were then challenged with iPer a 5 and bPer a 5 at 1.0 *μ*g/mL. The values shown are mean ± SEM for the sera from 4 different subjects. *P < 0.05 in comparison with the corresponding carrier alone control.

iPer a 5 and bPer a 5 at 1.0 *μ*g/mL induced approximately up to 4.5- and 3.2-fold increase in the expression of CD63 and CCR3 in CD63 and CCR3 double positive cells when incubating with passively sensitized basophils (by sera from American CR allergy). Both iPer a 5 and bPer a 5 had no effect on the basophils sensitized by the sera from HC (Figure 7).

## 4. Discussion

Aerosolized proteins derived from saliva, fecal material, secretions, cast skins, debris, and dead bodies of cockroaches induce IgE-mediated hypersensitivity [24]. To better understand the Per a 5 mediated CR allergies and promise to improve diagnosis and treatment of CR allergies, we prepared biologically active and highly pure American CR allergen Per a 5 in relatively large amount in the present study. We have identified Per a 5 as a novel American CR allergen, which is recognized by 25% of the subjects with American CR allergy.

Several allergens from German and American CR have been identified and their IgE cross-reactivity has been described. These include Bla g 1 and Per a 1 (food intake and digestion); Bla g 2 and Per a 2 (inactive aspartic protease); Bla g 3 and Per a 3 (arylphorin-like storage protein); Bla g 4 and Per a 4 (male pheromone transport lipocalin); and Bla g 7 and Per a 7 (tropomyosin) [25]. Among CR allergens, 3 GSTs were identified from male adults of German CR by glutathione-agarose affinity chromatography including one in which N-terminal amino acid sequence is identical to that of Bla g 5 [26]. Two IgE-reactive GSTs were detected from 25 IgE-reactive spots by proteomic analysis, and one of them was found to be Bla g 5 [27]. Recently, a Delta class GST (BgGSTD1), which has 15% amino acid sequence identity with Bla g 5, was purified from the German CR [28]. However, the allergenicity and IgE cross-reactivity of the GST homologues in American CR have not been reported. As a member of the Delta class GSTs, Per a 5 shows 81% sequence similarity to BGGSTD1, but only 13% similarity to Bla g 5, a Sigma class GST identified from German CR. In the present study, only 25% IgE reactivity to rPer a 5 was observed when sera from cockroach-sensitive patients was incubated with rPer a 5, suggesting that Per a 5 is not likely a major allergen in American CR. The IgE reactivity rate of Per a 5 seems lower than that of Bla g 5 (30 to 71%) [12–14], but higher than that of BGGSTD1 (17.9%) [28]. It has previously been reported that American CR extracts could not inhibit IgE antibody binding to Bla g 5 using an RIA assay, suggesting that there was no significant IgE cross-reactivity between GSTs from these two species [12]. However, Huang et al. found that GST(s) in American CR is(are) allergenic and is(are) pan-allergens in CR and mites [29]. The finding that Per a 5 reacts with specific IgE of American CR allergy in the present study suggests that GST in American CR is allergenic.

We prepared rPer a 5 by using two different expression systems in the present study and found that as little as 2 L of *E. coli* and Sf-9 cell culture medium was able to produce 8 and 16 mg of highly pure rPer a 5, respectively, which is enough for functional study of Per a 5. The *E. coli* system is a well-established system offering many advantages: easy handling of the bacteria cells and selection of a large variety of vectors using different promoters. Among the disadvantages, overexpressed proteins can be incorrectly folded and may require chemical refolding procedures to obtain the protein in a native, fully active, biological form. In the present study, we chose pCold II vector and *E. coli* Origami host strain to produce bPer a 5 in a soluble form without any reconstitution process. The eukaryotic baculovirus expression system is characterized by an extensive array of posttranslational processing, typical for higher eukaryotic cells. The production of recombinant proteins in this system offers the advantage that secreted proteins are often glycosylated and disulphide-bonded correctly leading to a biologically active conformation. Because of the advantage of baculovirus-infected insect cell expression system, we employed it to express iPer a 5 and managed to obtain substantial quantity of iPer a 5 in the present study. Numerous insect allergens such as Api m 1 [30], Api m 2 [31] from honeybee venom, Sol i 3 [32] from ant venom, Dol m 5 [33] from bald-faced hornet (*Dolichovespula maculate*), Cul s 1 [34] from the North American midge (*Culicoides sonorensis*), Der f 1 [35] from house dust mite, Blo t 11 [36] from dust mite (*Blomia tropicalis*), Lep d 2 [15] from dust mite (*Lepidoglyphus destructor*), and Aed a 1 and Aed a 2 [37] from mosquito (*Aedes aegypti*) have been successfully expressed in insect cells using a baculovirus expression system. They are reported to possess similar structural and biological activities to their natural forms [38].

Regarding the immunoreactivity, rPer a 5 produced in the two systems showed IgE-binding activities towards the sera from the American CR allergic patients. iPer a 5 seems to have better binding activity than bPer a 5, implicating that the IgE-binding activity of rPer a 5 is likely to associate with its glycosylation sites and tertiary conformation. One motif (198NHSG201) was predicted to be the glycosylation motif in Per a 5, but we are not sure of the numbers of *α*-Gals in it, as most of the carbohydrate epitopes are known to lead false-positive reactions except *α*-Gal [39]. The far UV CD spectra of recombinant iPer a 5 and bPer a 5 showed similar secondary structures, which consist of predominantly *α*-helical structure. But iPer a 5 appears to have more *α*-helical structures than bPer a 5. IgE-binding epitopes recognized by IgE antibodies are either linear or conformational and are located on the surface of the molecule accessible to antibodies [40]. Conformational epitope comprises amino acids that are close in space in the folded molecule, despite being noncontiguous in the amino acid sequence. They are dependent on the 3-dimensional structure of the protein. For globular inhaled allergens, conformational epitopes play a very important role in allergenicity [41, 42]. Therefore, it seems likely that extra conformational IgE-binding epitopes may exist in iPer a 5, as a result of more *α*-helical structures in its molecular structure.

The basophil activation test we employed herein is a more advanced technique for determination of allergenicity of a given compound. We confirm that Per a 5 is an active allergen of CR as it is able to activate CR-sensitized basophils. Similar to IgE-binding activity, iPer a 5 seems more potent in activation of basophils compared with bPer a 5, suggesting that the basophil-activating activity of rPer a 5 is likely to associate with its glycosylation sites and tertiary conformation. Obviously, further work is required to confirm our anticipation.

The availability of recombinant allergens has increased our understanding of IgE-mediated allergies and promises to improve diagnosis and treatment of these diseases [43]. In our case, rPer a 5 should be a useful tool for functional and clinical study. These observations also confirm that baculovirus-infected insect cell expression system is more suitable for the production of more active Per a 5 allergen than *E. coli* expression system. In conclusion, we have cloned and prepared two rPer a 5 allergens by using a eukaryotic and a prokaryotic expression system. We confirm that there is GST allergen (Per a 5) in American CR, though it is not a major allergen. rPer a 5 should be a useful tool for studying and understanding the role of Per a 5 in CR allergy.
